# Stereological Analysis of Liver Biopsy Histology Sections as a Reference Standard for Validating Non-Invasive Liver Fat Fraction Measurements by MRI

**DOI:** 10.1371/journal.pone.0160789

**Published:** 2016-08-08

**Authors:** Tim G. St. Pierre, Michael J. House, Sander J. Bangma, Wenjie Pang, Andrew Bathgate, Eng K. Gan, Oyekoya T. Ayonrinde, Prithi S. Bhathal, Andrew Clouston, John K. Olynyk, Leon A. Adams

**Affiliations:** 1 School of Physics, The University of Western Australia, Crawley, Western Australia, Australia; 2 School of Medicine and Pharmacology, The University of Western Australia, Crawley, Western Australia, Australia; 3 Department of Gastroenterology, Fremantle Hospital, Fremantle, Western Australia, Australia; 4 Liver Transplant Unit, Sir Charles Gairdner Hospital, Nedlands, Western Australia, Australia; 5 Resonance Health Ltd, Claremont, Western Australia, Australia; 6 Department of Pathology, The University of Melbourne, Melbourne, Victoria, Australia; 7 Faculty of Health Sciences, Curtin University of Technology, Bentley, Western Australia, Australia; 8 Centre for Liver Disease Research, School of Medicine Translational Research Institute, The University of Queensland, Woolloongabba, Queensland, Australia; 9 Institute for Immunology & Infectious Diseases, Murdoch University, Murdoch, Western Australia, Australia; Brandeis University, UNITED STATES

## Abstract

**Background and Aims:**

Validation of non-invasive methods of liver fat quantification requires a reference standard. However, using standard histopathology assessment of liver biopsies is problematical because of poor repeatability. We aimed to assess a stereological method of measuring volumetric liver fat fraction (VLFF) in liver biopsies and to use the method to validate a magnetic resonance imaging method for measurement of VLFF.

**Methods:**

VLFFs were measured in 59 subjects (1) by three independent analysts using a stereological point counting technique combined with the Delesse principle on liver biopsy histological sections and (2) by three independent analysts using the HepaFat-Scan^®^ technique on magnetic resonance images of the liver. Bland Altman statistics and intraclass correlation (IC) were used to assess the repeatability of each method and the bias between the methods of liver fat fraction measurement.

**Results:**

Inter-analyst repeatability coefficients for the stereology and HepaFat-Scan^®^ methods were 8.2 (95% CI 7.7–8.8)% and 2.4 (95% CI 2.2–2.5)% VLFF respectively. IC coefficients were 0.86 (95% CI 0.69–0.93) and 0.990 (95% CI 0.985–0.994) respectively. Small biases (≤3.4%) were observable between two pairs of analysts using stereology while no significant biases were observable between any of the three pairs of analysts using HepaFat-Scan^®^. A bias of 1.4±0.5% VLFF was observed between the HepaFat-Scan^®^ method and the stereological method.

**Conclusions:**

Repeatability of the stereological method is superior to the previously reported performance of assessment of hepatic steatosis by histopathologists and is a suitable reference standard for validating non-invasive methods of measurement of VLFF.

## Introduction

Fatty liver, particularly non-alcoholic fatty liver disease (NAFLD) is a common aberrant liver condition encountered in many general populations. The prevalence of NAFLD is considered to be increasing [1–4] and there is growing literature describing associations of NAFLD with cirrhosis [5], metabolic disorders, notably obesity [6], diabetes [7] and atherosclerotic cardiovascular disease [8, 9]. Consequently, there is a need for accurate quantification of liver fat in research settings. There is also likely to be increasing need in future clinical practice where accurate fat quantification could be used to improve liver resection strategies and transplant screening [10]. Such measurements could also become more relevant for diagnosing and monitoring the treatment of conditions like NAFLD and NASH in the advent of pharmaceutical treatments becoming available. An increasing number of published studies describe the correlation between measures of hepatic steatosis using magnetic resonance imaging (MRI) or magnetic resonance spectroscopy (MRS) methods and measures of hepatic steatosis assessed in liver biopsy specimens [11–31]. The interest in the correlation between the magnetic resonance (MR) measure and the histological measure stems from the fact that the vast majority of the gastroenterological and hepatological literature regarding relationships between degree of liver steatosis and progression of disease and patient outcomes use histological studies of liver biopsies to deduce the degree of steatosis. Hence the measurement of steatosis in histological sections of biopsies has been considered the reference standard measurement. Knowledge of how MR measures of liver steatosis relate to histological measures of steatosis is a requirement for interpreting the results from MR in the context of observations made from the vast literature of clinical studies using biopsy.

Moderate to strong correlations between MR measures and measures of steatosis in biopsy histological sections are found in the aforementioned studies. However, a strong correlation coefficient between a MR measurement of steatosis and measures of steatosis in biopsies is, in itself, not sufficient to enable MR results to be translated to biopsy results. The regression equation relating the MR measure to the biopsy measure is also required but is usually not reported. Such an equation enables the MR method to be calibrated against the biopsy method. In principle, once calibrated, the MR method can then be validated on other MR scanners and in different populations of patients by comparison with the reference standard, namely the measurement of steatosis in biopsies of those patients. A lack of significant bias between the MR method and the reference standard method would indicate validation of the calibration of the MR method in the new population.

Magnetic resonance methods tend to report the proton density fat fraction or the fraction of proton signal emanating from fat molecules while biopsy assessments often report a histopathologist’s semi-quantitative assessment of steatosis in terms of the fraction of hepatocytes involved with fatty vesicles. This difference in the nature of the two measurements leads to several problems for calibration and validation of the MR method against biopsy.

Firstly, histopathologists’ assessments of steatosis in liver biopsies have very poor reproducibility making them an unreliable reference standard [32]. Even in the case of accurate assessment by the histopathologist, MR reported and histology reported fat fractions will not be equivalent. For example, if a histopathologist reports 100% of hepatocytes being involved with steatosis, there will still be water protons present in the tissue and hence MR methods will report a fraction of protons that is substantially lower than 100%. Secondly, for a given fraction of hepatocytes involved with steatosis, the amount of fat per cell may vary between individuals and between forms of disease. For example, the ratio of microvesicular to macrovesicular fat may vary between patients. In some studies investigators have attempted to ameliorate the problem of the semi-quantitative nature of histopathological assessment by employing computer assisted morphometric methods to measure the fractional area of the tissue in a biopsy specimen that is fat [14, 18, 19, 25, 26, 29]. The fractional area of the thin tissue section that is represented by fatty vesicles is equivalent to the volumetric fraction of fatty vesicles in the tissue (the Delesse Principle) [33]. However, computer assisted morphometric methods rely on assumptions regarding the shape and size of fatty vesicles and also rely on a subjective determination of image intensity thresholds [14, 19, 25, 26, 29]. As such, computer assisted morphometric analysis methods of measuring the fraction of fat in the liver are subject to a bias, the magnitude of the bias depending on the assumptions used. Magnetic resonance methods of measuring the fraction of protons that are from fatty molecules are also subject to bias. Different methods can yield different results for the same liver tissue, the results depending on whether or not background noise is taken into account, and the value of various MR data acquisition parameters used, such as flip angle and repetition time [34–36].

As such, there is a need for a reliable reference standard against which non-invasive methods of measuring liver fat fraction can be evaluated that provides a quantitative link between the MR method and observations made on liver biopsy sections. Given that clinical guidelines related to the interpretation of the severity of hepatic steatosis (fatty liver) are based on hepatologists’ interpretations of liver biopsy specimens, there is a strong argument that reliable unbiased quantitative assessment of the fraction of fat in liver biopsy specimens should be the basis for an appropriate reference standard.

Stereological analysis of histological sections has been used for several decades to obtain estimates of the volume fraction of phases or materials in both biological tissues and inorganic media with minimum bias [33, 37–40]. In the current study, stereological analysis of liver biopsy histological sections was used to obtain volumetric liver fat fractions (here defined as the volume fraction of the liver that comprises fatty vesicles) in order to assess the bias in an MRI method for measuring volumetric liver fat fraction. The MRI method, HepaFat-Scan^®^, received FDA clearance for marketing in December 2013 and CE Mark in July 2014 and reports volumetric liver fat fraction rather than the usually reported proton density fat fraction.

## Materials and Methods

### Ethics Statement

Written informed consent was obtained from each subject and the study protocol conformed to the ethical guidelines of the 1975 Declaration of Helsinki. This study was approved by the Fremantle Hospital Human Research Ethics Committee and the Sir Charles Gairdner Hospital Human Research Ethics Committee.

### Subjects

65 patients were enrolled in the study. The patients were recruited from the hepatology outpatient clinics at Fremantle and Sir Charles Gairdner Hospitals, Western Australia. The patient inclusion criteria were: age between 18 and 75 years, requirement of a liver biopsy for routine clinical management, and written informed consent. Exclusion criteria were: contraindications for MRI, pregnancy or lactation. One patient was excluded due to fluctuations in weight and alcohol consumption during the period between liver biopsy and MRI. An additional five patients were excluded for incorrect MRI data acquisition or unavailability of histological slides for analysis leaving 59 participants. The median interval between biopsy and MRI was 57 days. Summary data for the subjects are shown in Table 1. The diversity of etiologies included in the study was to ensure that volumetric liver fat fractions across the entire clinically encountered range were represented in the study.

**Table 1:** Study cohort clinical data.

Characteristics	Patients Recruited	Patients with MRI and Biopsy Histology Results
N	65	59
Gender (F/M)	31/34	29/30
Age (years), median (range)	56 (20–72)	56 (20–72)
BMI (kg/m^2^), mean ± st. dev.	29.00 ± 5.11	28.92 ± 5.17
LIC (mg/g), median (range)	0.9 (0.3–4.8)	0.9 (0.3–4.4)
**Diagnosis**		
AIH	3	3
ALD	3	2
HBV-HCV	18	16
NAFLD	11	10
NASH	19	17
NORM	3	3
PSC	4	4
OTHER	4	4

Abbreviations: AIH, autoimmune hepatitis; ALD, alcoholic liver disease; BMI, body mass index; HBV-HCV, viral hepatitis B/C; LIC, liver iron concentration; MRI, magnetic resonance imaging; NAFLD, non-alcoholic fatty liver disease; NASH, nonalcoholic steatohepatitis; NORM, normal; PSC, primary sclerosing cholangitis.

### Measurement of Volumetric Fat Fraction in Liver Histological Sections

The patients underwent percutaneous liver biopsy with ultrasound guidance as part of their routine clinical management. Biopsy sections were prepared and stained with Masson’s trichrome. Histological sections of the biopsies were scanned in colour using an Aperio ScanScope XT (Aperio Technologies, Inc., California, USA) automated slide scanner and ImageScope software. The mean area of biopsy tissue in the histological sections was 14.4 (±SD 6.2) mm^2^.

**Fig 1:**
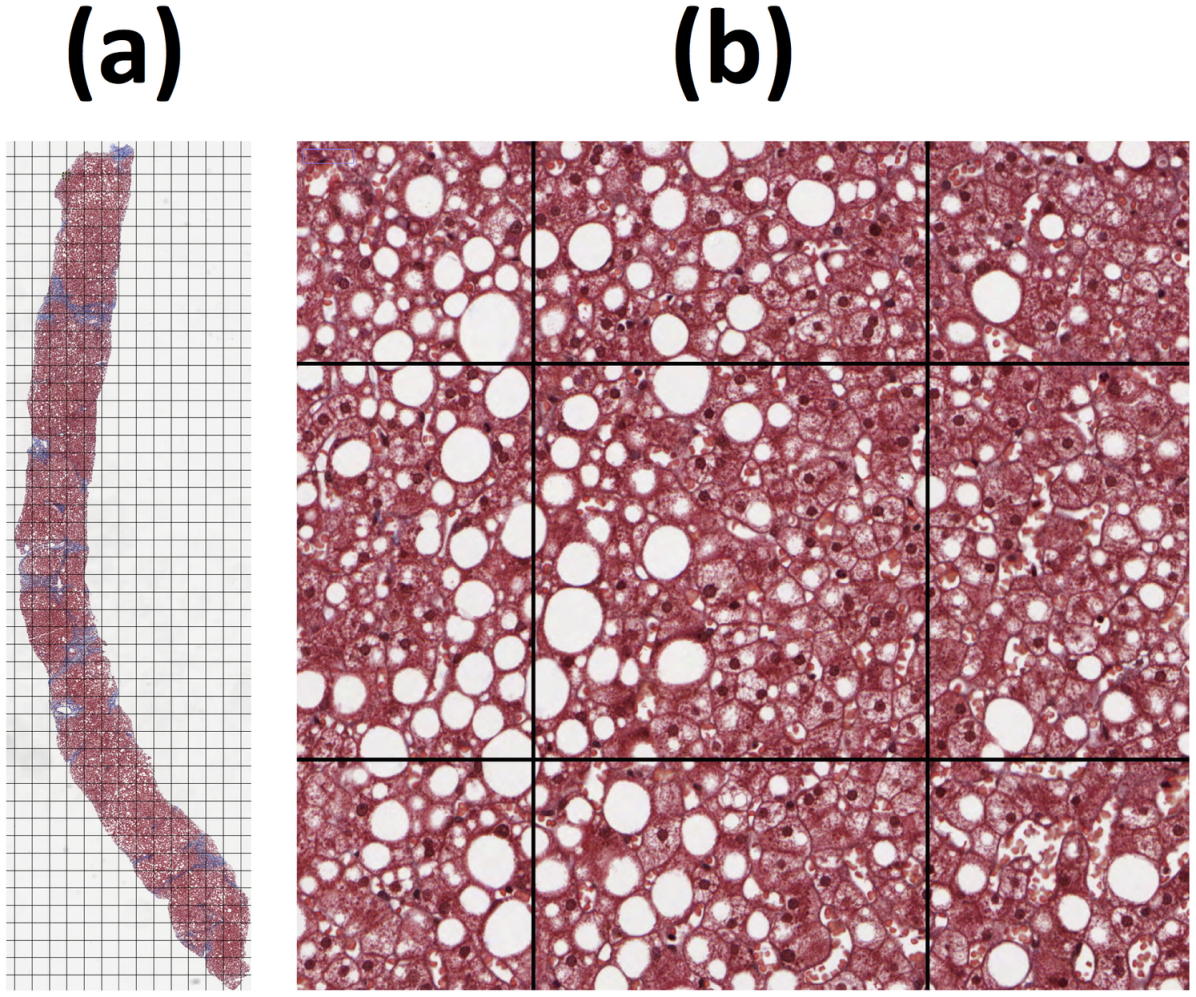
Stereological analysis of biopsy sections. (a) Example histological section of a liver biopsy with square grid randomly placed over image. The grid size was adjusted so that approximately 200 intersections are located within the tissue boundary. Every intersection within the tissue boundary is visually examined to determine whether or not it falls on a fat vesicle. The volumetric fat fraction for this example was determined to be 18.6%. (b) High magnification image of four of the intersections in (a). The lower left grid intersection was assessed to be within a fat vesicle while the other three intersections were assessed to be outside fat vesicles. The distance between two neighbouring intersections is 250 microns or 504 pixels in this example. Note that the grid lines are shown thicker here than in the analysis for clarity of display. During analysis the grid lines are one pixel wide at the highest magnification.

Volumetric fat fraction was measured from the histological thin sections using the stereological grid-point counting method combined with the Delesse principle [33, 40]. The key reasons for choosing this method are that such an approach (1) measures volumetric fat fraction (which can be compared with the HepaFat-Scan^®^ MRI measurement) and (2) in principle is unbiased with regard to the methodology [41, 42]. The stereological method of grid-point counting to measure the fat content of liver biopsies has been used in previous studies [37–39, 43]. The method is based on systematic random sampling of the microscope digital image. The random component of the sampling is the random placement of a square grid over the image while the systematic component is assessment of every grid intersection that falls on the tissue section. Each intersection is examined by an analyst to determine whether or not it falls on a fat vesicle (Fig 1). There are no assumptions about size, shape, or distribution of fat vesicles in the sample. There is an assumption that if there is tissue shrinkage or expansion, that the holes representing the fat vesicles shrink or expand to the same degree.

For each biopsy digital image, the mesh of the grid was adjusted so that approximately 200 intersections fell within the tissue boundaries. The number of 200 intersections was determined as a compromise between precision of estimate and time required to complete an analysis as follows. Navigation to, examination of, and recording of the result from an intersection requires approximately 1 minute. The standard error on the estimate of area fraction, *f*, using the stereological technique with *n* intersections is given as √[*f*(1-*f*)/*n*] [40]. Initial semi-quantitative examination of the biopsy sections indicated that vesicle area fractions ranged from approximately 1% to 40%. The choice of 200 intersections per biopsy results in standard errors ranging from 0.7% for volumetric fat fractions of 1% at the low end of the range up to 3% for volumetric fat fractions of 40% at the high end of the range with an overall analyst observation time of approximately 3 hours per biopsy. With an average standard error on a measurement of *f* of approximately 2% and 59 biopsies, 200 intersections per biopsy would result in a lower limit of detection of bias of stereology against another method of approximately 0.02/√59 = 0.3% volumetric liver fat fraction.

Each intersection was inspected and assigned to be either within a fat vesicle, outside of a fat vesicle, or sitting on the boundary of a fat vesicle. After initial assignment of each intersection, 50% of the intersections initially assigned to boundaries were reassigned to being inside a vesicle while the other 50% were reassigned to being outside a vesicle. The area fraction of the fat vesicles was estimated by calculating the ratio of intersections assigned to being inside fat vesicles to the total number of intersections within the tissue boundaries.

Each biopsy sample was analysed using the point counting stereology method by three independent analysts.

### Grading of Steatosis in Biopsy Sections by Hepatopathologists

Three experienced hepatopathologists (blinded to the patients’ identities, the MRI results, and the stereology results) graded each biopsy sample from 0 to 3 according to the NASH Clinical Research Network Scoring System [44]. Receiver operating characteristic curve (ROC curve) analysis was used to identify thresholds of volumetric liver fat fraction by stereology and by MRI that resulted in the highest sum of sensitivity and specificity for prediction of steatosis grades >0, >1, and >2 for each hepatopathologist.

### MRI Data Acquisition

All MRI measurements were made on Siemens 1.5 T Avanto scanners (Siemens Medical Systems, Erlangen, Germany) at Fremantle Hospital, St John of God Murdoch Hospital, and Hollywood Private Hospital, Western Australia. The median time between biopsy and MRI was 57 days. Data were acquired as prescribed by the HepaFat-Scan^®^ methodology (Resonance Health Ltd, Perth, Australia). Phased-array torso coils were centred over the liver of the subjects. MRI data acquisition comprised an opposed-phase, in-phase, opposed-phase gradient echo sequence (TEs 2.38, 4.76, 7.14 ms, TR 88 ms, 1 excitation, flip angle 70 degrees, bandwidth 500 Hz). Data from three axial slices, positioned through the widest part of the liver, were acquired in a single breath-hold. The slice thickness was 4 mm and the matrix was 256 x 256 with a field of view 300 x 300 mm. Liver iron concentrations (LIC) were measured using a validated non-invasive MRI method (FerriScan^®^) [45, 46].

### MR Image Processing

**Fig 2:**
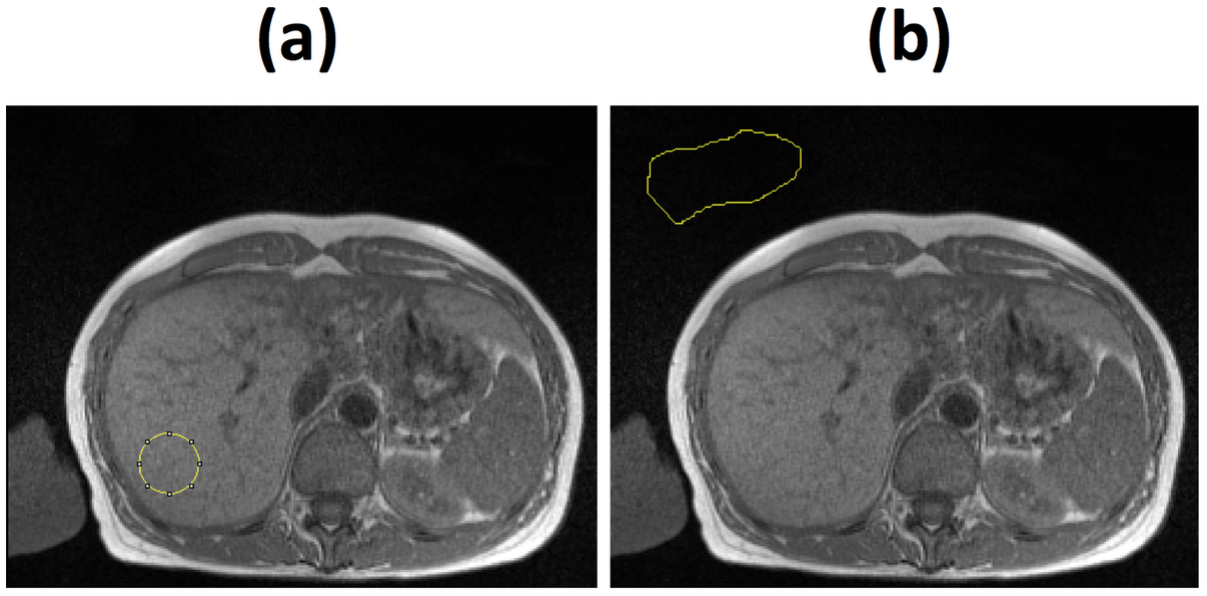
Magnetic resonance images of the liver. (a) A typical region of interest selected for analysis within the liver magnetic resonance image. (b) A typical region of interest of free space selected within the MR image for measurement of background noise.

On each of the three MR image slices, a circular region of interest (ROI) about 580 mm^2^ was delineated within the right lobe of the liver, avoiding large intrahepatic vessels and any obvious motion-affected regions (Fig 2a). The image intensity was measured in the ROI and also in a region of free space outside of the patient in order to measure background noise (Fig 2b). The data from these measurements were then processed by the HepaFat-Scan^®^ software (Resonance Health Analysis Services Pty Ltd, Claremont, WA, Australia) to generate a volumetric liver fat fraction. Each image dataset was analysed by three independent analysts.

### Statistical Analysis

Repeatability coefficients for each method of measurement of volumetric liver fat fraction were calculated from the three measurements on each subject by three different analysts. Firstly the within-subject standard deviation, *s*_*w*_, was calculated by taking the square root of the residual mean square obtained from one-way analysis of variance, with the subject as the factor [47]. The repeatability coefficient for each method is given by 1.96(√2) *s*_*w*_ or 2.77 *s*_*w*_. In the context of this study, two readings by two different analysts working on the same biopsy section or MR image data will be within 2.77 *s*_*w*_ of each other for 95% of subjects. The upper and lower 95% confidence limits on the calculated repeatability coefficients were calculated following the method of Bland and Altman [47]. The conformity of analysts using each technique was also assessed by calculating the intraclass correlation coefficient (ICC). The method of Shrout and Fleiss [48] was used to calculate the ICCs (type (2,1)) together with their 95% confidence intervals. The ICC is an estimate of the proportion of the total variance of all subjects and all analysts that is due to the subjects alone, the remaining variance being due to other sources such as inter-analyst and intra-analyst variability.

For the stereological measurement of volumetric liver fat fraction in biopsies, the MR measurement of volumetric liver fat fraction by HepaFat-Scan^®^, and the assessment of percentage fat by the hepatopathologists, the bias between the different pairs of analysts was assessed using the method of Bland and Altman [47].

The degree of bias between measurements of volumetric liver fat fraction in the biopsy sections and measurements of volumetric liver fat fraction made by HepaFat-Scan was determined using the methods of Bland and Altman [47]. The average measurement from three independent analysts was used for both the biopsy measurement and the HepaFat-Scan measurement. The 95% limits of agreement between the two methods of measuring volumetric liver fat fraction were also assessed using the methods of Bland and Altman [47].

## Results

### Demographic and Clinical Data

Demographic and clinical data for the recruited patients are shown in Table 1. The distribution of METAVIR fibrosis stages for the patients included in the study was F0 (19%), F1 (38%), F2 (22%), F3 (12%), F4 (9%). The distribution of steatosis grades according to the NASH CRN grading system [44] as assessed by the average of the three percentage steatosis assessments by the histopathologists was: grade 0 (47%), grade 1 (15%), grade 2 (17%), grade 3 (20%). Five patients (9%) had liver iron concentration (LIC) levels above the upper 95% limit of normal (1.8 mg Fe/g dry tissue) with the maximum LIC being 4.4 mg Fe/g dry tissue.

The volumetric liver fat fractions determined by stereological analysis of the biopsy sections ranged from 0.7% to 32.6% while those measured by HepaFat-Scan^®^ ranged from 0.8% to 32.7%. Details of all data reported in this study are shown in S1 Table.

### Inter-Analyst Repeatability of Measurements of Fat Fraction

The repeatability coefficient (three analysts) of the grid point counting stereological method for measurement of volumetric fat fractions in the biopsy sections was found to be 8.2 (95% CI 7.7–8.8) % volumetric fat fraction indicating that 95% of pairs of results from any pair of analysts were within 8.2% of each other. The repeatability coefficient (three analysts) of HepaFat-Scan^®^ for measurement of volumetric fat fractions was found to be 2.4 (95% CI 2.2–2.5) % volumetric fat fraction indicating that 95% of pairs of results from any pair of analysts were within 2.4% of each other. The repeatability coefficient (three hepatopathologists) for assessment of percentage steatosis was found to be 38 (95% CI 35–40) % indicating that 95% of pairs of results from any pair of hepatopathologists were within 38% of each other.

The ICC determined from the stereological analyses of the 59 biopsies by three analysts was 0.86 (95% CI 0.69–0.93). The ICC determined from the analyses of the 59 sets of MRI data by three analysts using the HepaFat-Scan^®^ method was 0.990 (95% CI 0.985–0.994). The ICC determined from the hepatopathologists assessments of percentage steatosis in the 59 biopsies was 0.79 (95% CI 0.68–0.87).

**Fig 3:**
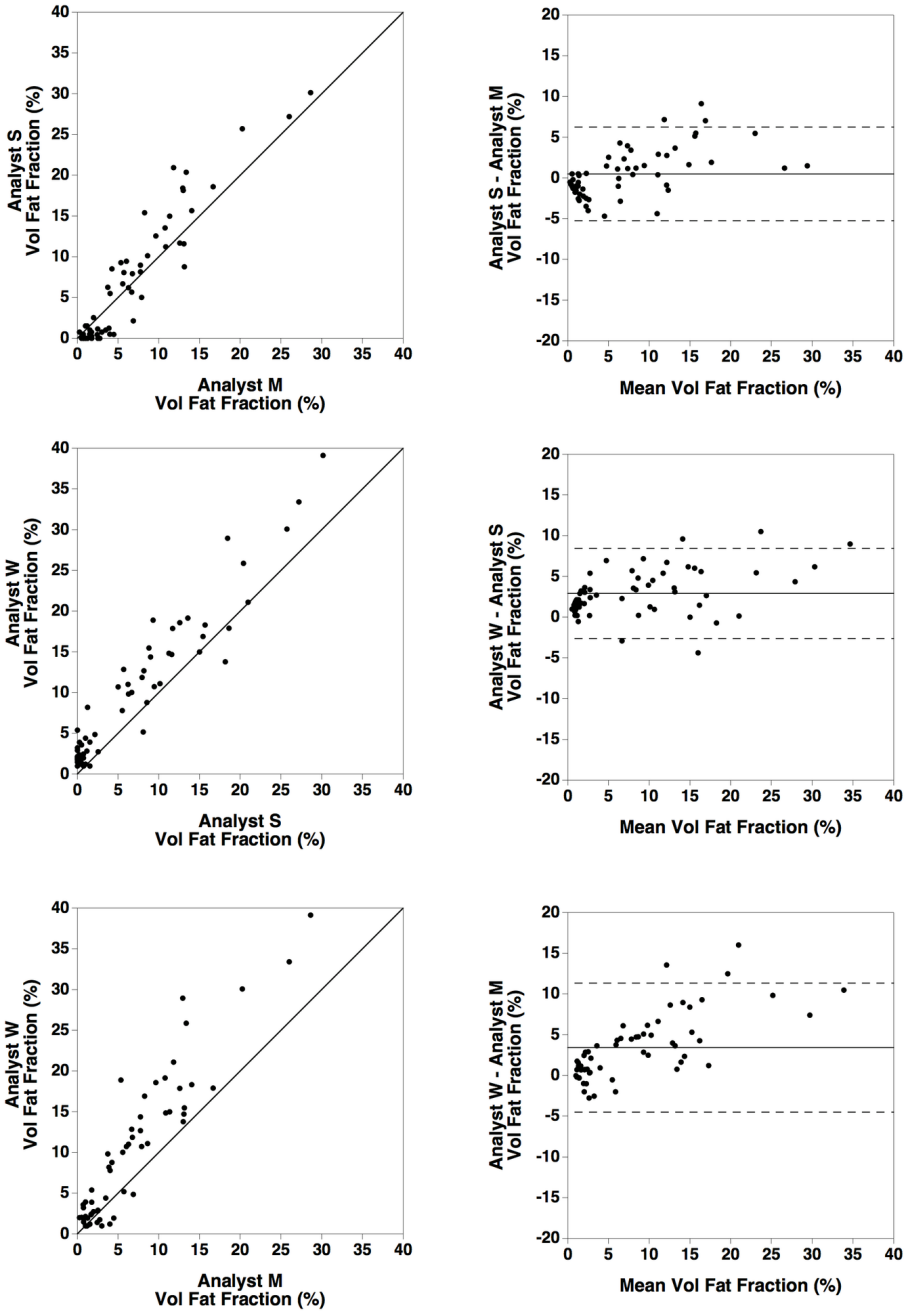
Inter-analyst comparison of the stereology results. Comparisons of results of measurement of volumetric liver fat fraction in 59 biopsy sections by three independent analysts (identified as S, M, and W) using the stereology method. The left column shows plots of the results from one analyst against the results from another. The solid line is the line of equivalence (not a line of best fit). The right columns shows Bland Altman plots of the difference in results between two analysts plotted against the mean result from two analysts. The horizontal solid line indicates the mean difference between the two analysts while the dashed lines indicate the upper and lower 95% limits of agreement between the two analysts.

Assessment of the bias between the three analysts using stereology to measure the volumetric liver fat fraction in the biopsy sections indicated that there was no significant bias between two of the analysts (Analysts S and Analyst M) but that there was significant bias between Analyst W and the other two analysts 2.9 ± 0.4% and 3.4 ± 0.5% (Fig 3)

**Fig 4:**
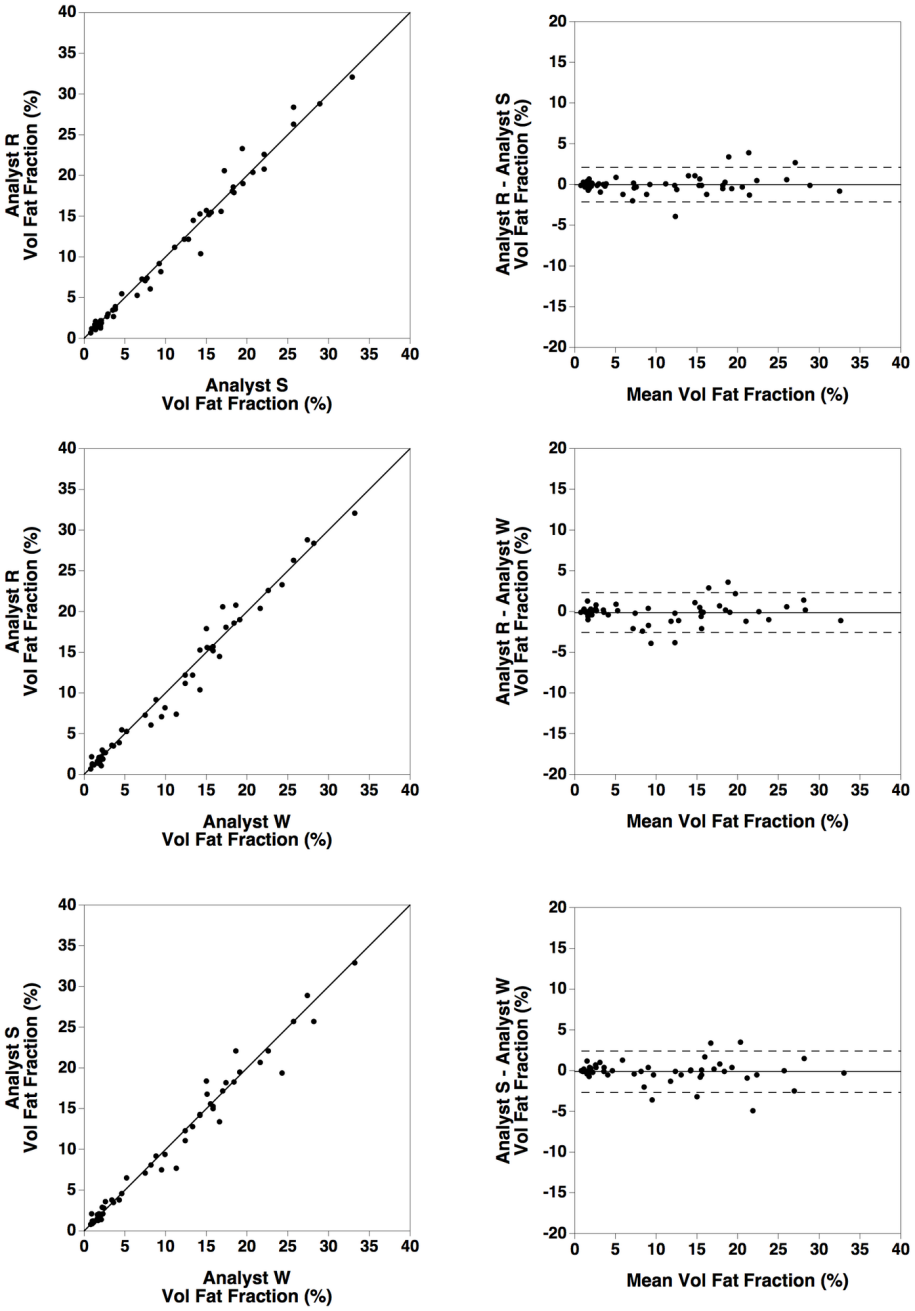
Inter-analyst comparison of the HepaFat-Scan^®^ MRI results. Comparisons of results of measurement of volumetric liver fat fraction in 59 MRI datasets by three independent analysts (identified as R, S, and W) using the HepaFat-Scan^®^ method. The left column shows plots of the results from one analyst against the results from another. The solid line is the line of equivalence (not a line of best fit). The right columns shows Bland Altman plots of the difference in results between two analysts plotted against the mean result from two analysts. The horizontal solid line indicates the mean difference between the two analysts while the dashed lines indicate the upper and lower 95% limits of agreement between the two analysts.

Assessment of the bias between the three analysts using HepaFat-Scan^®^ to measure the volumetric liver fat fraction indicated that there was no significant bias between any of the analysts (Fig 4).

**Fig 5:**
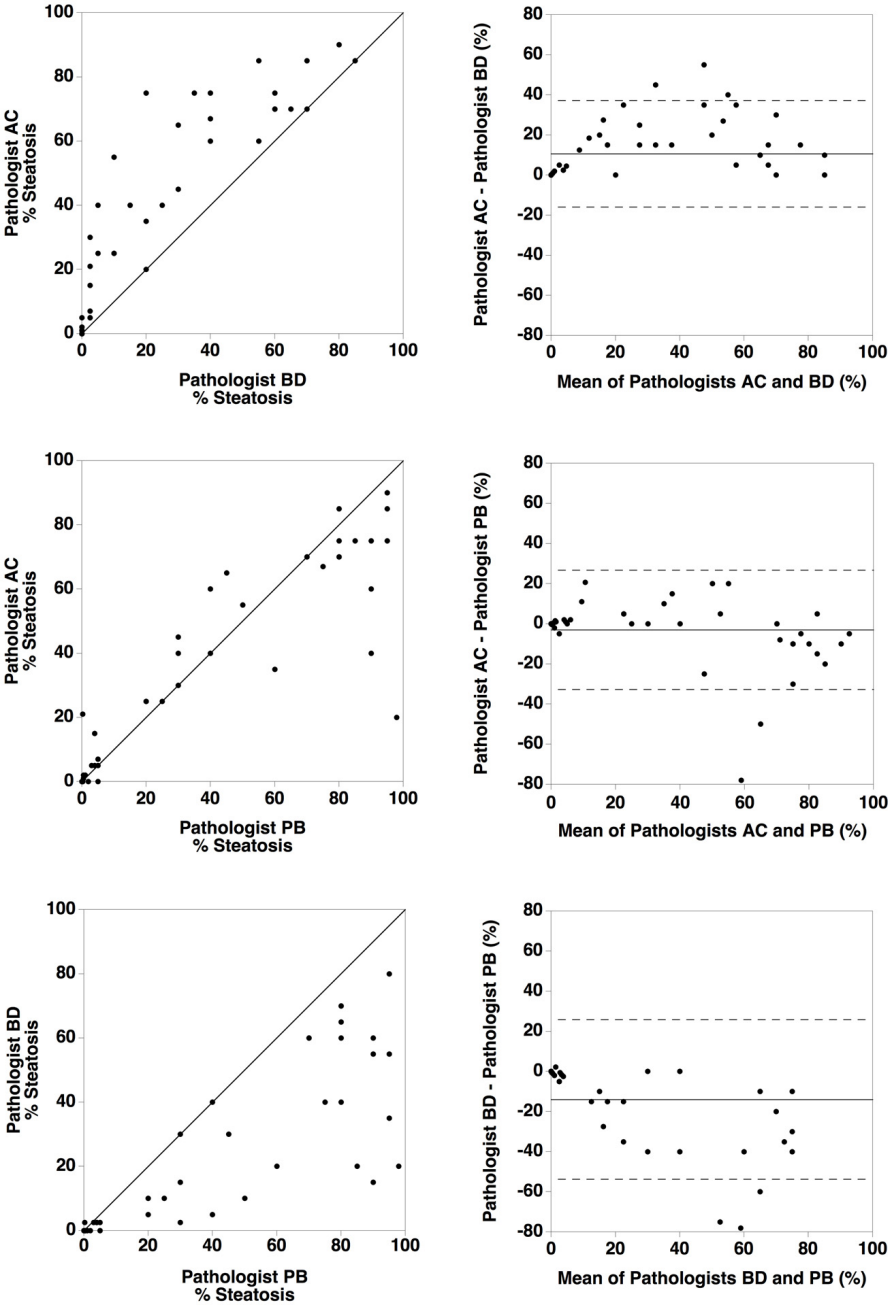
Inter-analyst comparison of hepatopathologists results. Comparisons of results of assessment of percentage steatosis in 59 biopsy sections by three independent hepatopathologists (identified as AC, BD, and PB). The left column shows plots of the results from one hepatopathologist against the results from another. The solid line is the line of equivalence (not a line of best fit). The right columns shows Bland Altman plots of the difference in results between two hepatopathologists plotted against the mean result from two hepatopathologists. The horizontal solid line indicates the mean difference between the two hepatopathologists while the dashed lines indicate the upper and lower 95% limits of agreement between the two hepatopathologists.

Measurement of the bias between the three hepatopathologists assessments of percentage steatosis in the biopsy sections indicated that there was no significant bias between two of the hepatopathologists (AC and PB) but that there was significant bias between hepatopathologist BD and the other two hepatopathologists 10.7 ± 1.8% and 13.9 ± 2.7% (Fig 5).

### Comparison of MRI and Histological Volumetric Fat Measurements

**Fig 6:**
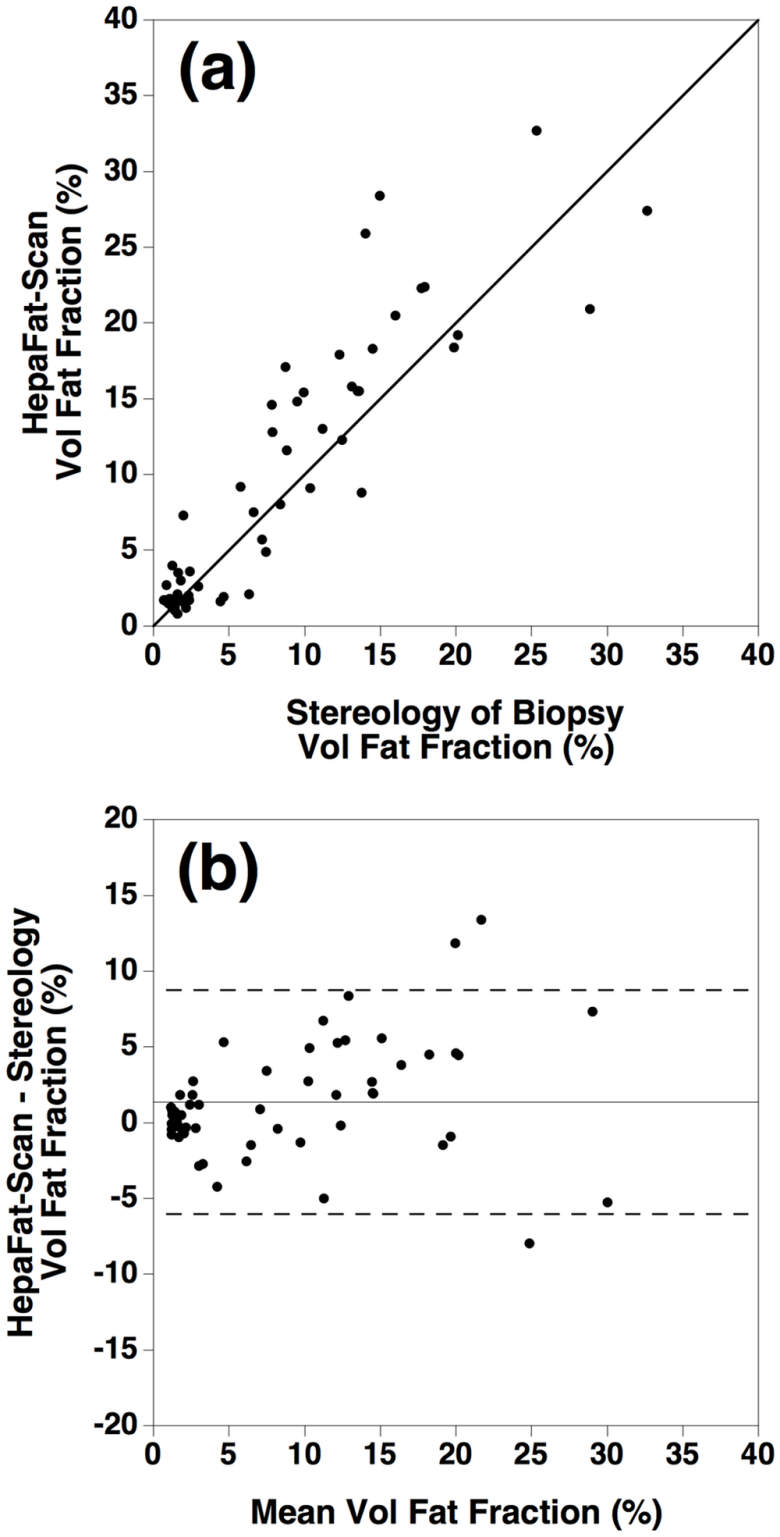
Comparison of volumetric liver fat fraction measured by HepaFat-Scan^®^ and stereology. (a) Volumetric liver fat fraction measured by HepaFat-Scan^®^ versus the volumetric liver fat fraction measured by stereology from histology sections of liver biopsy samples. The solid line is the line of equivalence (not a line of best fit). (b) Bland Altman plot showing the differences of the volumetric liver fat fractions measured by HepaFat-Scan^®^ and by stereological analysis of liver biopsy histology sections plotted against mean of the two measurements. The solid line indicates the mean difference while the dashed lines indicate the upper and lower 95% limits of agreement between the two measurements.

Fig 6a shows the volumetric liver fat fractions measured by MRI (mean of three observers) versus the volumetric liver fat fractions measured from the histological biopsy sections (mean of three observers) for the 59 subjects. The solid line is the line of equivalence. A Bland Altman plot of the difference between the two measurements plotted against the mean of the two measurements of volumetric liver fat fraction is shown in Fig 6b. The mean difference of the volumetric fat fractions measured by the two techniques was found to be 1.4% ± SE 0.5% (solid line in Fig 6b). The upper and lower 95% limits of agreement between the two techniques were found to be 8.7% and -6.0% respectively (dashed lines in Fig 6). No significant difference was found between the mean difference for the 5 subjects with elevated LIC and the mean difference for all other subjects. The subject with the highest LIC had the smallest bias relative to the non-iron-loaded subjects. In order to assess any impact of fibrosis stage on the bias and precision observed between the MRI and stereology measurements of volumetric liver fat fraction, VLFF differences were grouped into five categories corresponding to the METAVIR fibrosis stages 0 to 4. One way ANOVA showed neither significant differences among the mean differences (p = 0.80) nor significant differences among the variances of differences (p = 0.18) for each fibrosis stage.

### Comparison of MRI and Stereology with Histopathology Grading

Table 2 shows the optimal cut points (thresholds) of volumetric liver fat fraction (as a percentage) measured by stereology and by HepaFat-Scan^®^ for discrimination of the different grades of steatosis assessed by the three hepatopathologists. Table 2 also shows the areas under the ROC curves and sensitivities and specificities of stereology and HepaFat-Scan^®^ for predicting steatosis grades assessed by each of the three hepatopathologists using the stated cut points.

**Table 2:** Receiver operating characteristic curve analysis. Receiver operating characteristic curve analysis of stereology (3 analysts) and HepaFat-Scan (3 analysts) for prediction of hepatopathologists’ steatosis gradings in biopsies.

	Grade 0 vs Grades 1–3		Grades 0&1 vs Grades 2&3		Grades 0–2 vs Grade 3	
Stereology						
Stereology Cut Point (%)						
Pathologist AC	> 4.54		> 6.90		> 12.8	
Pathologist BD	> 5.20		> 11.8		> 16.9	
Pathologist PB	> 6.48		> 7.64		> 12.8	
AUROC (+/- SE)						
Pathologist AC	0.941	(0.033)	0.981	(0.013)	0.978	(0.019)
Pathologist BD	0.984	(0.013)	0.968	(0.020)	0.986	(0.016)
Pathologist PB	0.950	(0.028)	0.981	(0.014)	0.969	(0.021)
Sensitivity (%) (95% conf int)						
Pathologist AC	88.6	(73.3% to 96.8%)	100.0	(85.8% to 100.0%)	92.9	(66.1% to 99.8%)
Pathologist BD	100.0	(87.7% to 100.0%)	93.3	(68.1% to 99.8%)	100.0	(39.8% to 100.0%)
Pathologist PB	84.9	(68.1% to 94.9%)	100.0	(83.9% to 100.0%)	81.3	(54.4% to 96.0%)
Specificity (%) (95% conf int)						
Pathologist AC	95.8	(78.9% to 99.9%)	88.6	(73.3% to 96.8%)	95.6	(84.9% to 99.5%)
Pathologist BD	90.3	(74.2% to 98.0%)	93.2	(81.3% to 98.6%)	94.6	(84.9% to 98.9%)
Pathologist PB	96.2	(80.4% to 99.9%)	91.4	(76.9% to 98.2%)	100.0	(91.2% to 100.0%)
HepaFat-Scan						
HepaFat-Scan Cut Point (%)						
Pathologist AC	> 3.55		> 10.40		> 15.45	
Pathologist BD	> 5.30		> 15.45		> 20.70	
Pathologist PB	> 3.55		> 10.40		> 12.55	
AUROC (+/- SE)						
Pathologist AC	0.945	(0.031)	0.982	(0.013)	0.982	(0.014)
Pathologist BD	0.988	(0.012)	0.967	(0.023)	0.968	(0.023)
Pathologist PB	0.960	(0.033)	0.996	(0.005)	0.977	(0.016)
Sensitivity (%) (95% conf int)						
Pathologist AC	91.4	(76.9% to 98.2%)	91.7	(73.0% to 99.0%)	92.9	(66.1% to 99.8%)
Pathologist BD	100.0	(75.3% to 100.0%)	93.3	(68.1% to 99.8%)	100.0	(39.8% to 100.0%)
Pathologist PB	96.7	(82.8% to 99.9%)	100.0	(83.9% to 100.0%)	100.0	(79.4% to 100.0%)
Specificity (%) (95% conf int)						
Pathologist AC	91.4	(78.9% to 99.9%)	97.1	(85.1% to 99.9%)	93.3	(81.7% to 98.6%)
Pathologist BD	93.6	(78.6% to 99.2%)	95.5	(84.5% to 99.4%)	94.6	(84.9% to 98.9%)
Pathologist PB	92.3	(74.9% to 99.1%)	97.1	(85.1% to 99.9%)	90.0	(76.3% to 97.2%)

AUROC: Area under receiver operating characteristic curve

## Discussion

Technological advances in medical imaging have led to the development of a number of different non-invasive methods for quantifying hepatic steatosis [49–53]. Limitations of the use of conventional histopathological assessment of biopsy data for validation of or comparison with these methods are the variability and sensitivity of assessment [32]. For example, in a study of the assessment of steatosis in 46 biopsies by four expert pathologists from 4 prominent centers in 3 countries across Europe and North America, quantification of hepatic steatosis was found to be strongly observer-dependent, not reproducible, and did not correlate with the computerized estimation [32]. The ICC for the steatosis assessments made by the four pathologists on the 46 biopsies was 0.57 [32]. The ICC for steatosis assessments made by the three hepatopathologists on 59 biopsies in this study was 0.79 (95% CI 0.68–0.87) with a repeatability coefficient of 38 (95% CI 35–40) % steatosis. By contrast, the ICC determined from the current study using stereological measurement on 59 biopsies by three analysts was 0.86 (95% CI 0.68–0.93) with a repeatability coefficient of 8.2 (95% CI 7.7–8.8) % volumetric fat fraction. The results from this study demonstrate that stereological measurement of volumetric liver fat fraction in biopsies is a method of quantifying hepatic steatosis with a conformity that is superior to previously published studies on the performance of histopathological grading by visual assessment and a repeatability coefficient superior to that observed for the three hepatopathologists in the current study. The good repeatability of the stereological method together with the minimal bias associated with the principles of stereology indicate that this method could be considered as a new reference standard against which new technologies for measurement of liver fat fraction could be compared.

The patient population in the study had a wide range of hepatic steatosis and represents the full range of liver conditions encountered in hepatology clinics. All stages of liver fibrosis are represented in the patient cohort and no confounding effect of fibrosis on the volumetric liver fat fraction measurements could be detected. No confounding effects of LIC were detected either, most likely because the HepaFat-Scan^®^ technique accounts for T2* decay. However, only low to moderate iron loadings are represented in this study population (maximum LIC encountered was 4.4 mg Fe/g dw). High LIC has the potential to degrade accuracy of MR measurements of liver fat. Administration of T1 contrast agents such as gadolinium during imaging would also confound the measurement of fat fraction measured by HepaFat-Scan owing to the change in T1 of the non-fatty liver tissue.

The inter-analyst repeatability for the stereological measurements of volumetric liver fat fraction in the biopsy sections, although not as good as the repeatability observed for the HepaFat-Scan^®^ measurements, was still much better than that found for the three hepatopathologists in this study and the reported reproducibility of percentage steatosis by histopathologists in previous studies (compare Fig 3 in this report with Fig 1 in reference [32], for example). While significant biases were observed between some pairs of analysts using the stereological method, they were sufficiently small (≤ 3.4% volumetric fat fraction) compared with variations between the hepatopathologists (≤ 13.9% steatosis) to suggest that stereological analysis of liver biopsy sections is a better reference standard for measurement of fat fraction than hepatopathologists’ assessments (compare Figs 3 and 5).

The inter-analyst repeatability coefficient for measurement of volumetric liver fat fractions using HepaFat-Scan^®^ were significantly better than those for the stereological measurements (compare Figs 3 with 4) with no significant bias observable between the three analysts.

The upper and lower 95% limits of agreement between the measurements made by HepaFat-Scan^®^ and the stereological measurements on biopsy sections are determined both by the random errors on HepaFat-Scan^®^ and the random errors on the stereology measurements. While, in principle, stereological point counting analysis yields unbiased results [33], the precision of stereological analysis depends on the number of intersections examined. For a given volumetric fat fraction, *f*, precision improves with the square root of the number, *n*, of intersections examined [40]. Hence the choice of mesh size of the square grid (which determines the number of intersections to be examined) is a compromise between precision and the amount of analyst time require to complete examination of all intersections. With 200 intersections per biopsy, we found the time for completing all 200 intersection observations was approximately 3 hours per biopsy. Given the time required to accurately perform the stereology, it is unlikely such a technique would be suitable for routine clinical analysis.

The bias of 1.4 ± 0.5% volumetric fat fraction between the measurements made by HepaFat-Scan^®^ and the stereological measurements on biopsy sections is unlikely to be clinically significant. The magnitude of the bias is less than 5% of the overall range of volumetric liver fat fractions encountered in the population. Furthermore, having been measured, the bias can be taken into account when comparing other measurement techniques that might be compared against the stereologically measured reference standard in the future.

The areas under the ROC curves and sensitivities and specificities for prediction of the hepatopathologists’ steatosis grade assessments (Table 2) are also determined to an appreciable extent by the precision of the hepatopathologists. Nevertheless, the areas under the ROC curves and sensitivities and specificities indicate that both HepaFat-Scan^®^ and stereology could be used for grading steatosis. However, the cut points shown in Table 2 are specific to each hepatopathologist because of the biases that can occur between hepatopathologists as also shown by El-Badry et al [32].

A limitation of the current study is that only one form of histological staining was assessed (Masson’s trichrome). It is possible that different stains could affect the degree of bias measured. With the paraffin embedded Masson trichrome stained sections used in this study, lipid fat vesicles appear as holes or, if smaller than the thickness of the section, lower contrast vesicles. Other stains such as Oil Red can be used to directly stain lipid droplets on sections of frozen tissue [39] enabling computer assisted measurement of lipid vesicles using coloured pixel counting. While lipid staining techniques have a clear advantage over trichrome stained methods when using computer assisted pixel colour measurement, for point counting stereological techniques, where human observation is directed to a discrete number of exact points in an image for categorization, the advantage of lipid staining is less clear. For example, in the study by Catta-Preta and colleagues [39] comparing different staining techniques with a stereological point counting method for quantifying hepatic steatosis in mouse livers, hematoxylin and eosin (H&E) staining was found to give significantly better inter-observer reproducibility than Oil Red stained sections. Coefficients of variation of results from three analysts were found to be 4.0 and 27.3% (for mice on standard chow) and 1.2 and 7.5% (for mice on high fat chow) for H&E stained and Oil Red stained sections respectively.

In summary, the data presented here suggest that stereological analysis of liver biopsy sections using the point counting method together with the Delesse principle provides a good reference standard against which non-invasive methods of measurement of liver fat fraction can be compared or validated. In the case of this study, the MRI technique HepaFat-Scan^®^ has been shown to have minimal bias in the measurement of volumetric liver fat fraction when compared with biopsy measurements using stereology.

## Supporting Information

S1 Table Data reported in this study. (XLSX)
